# The lay health worker–patient relationship in promoting pulmonary rehabilitation (PR) in COPD: What makes it work?

**DOI:** 10.1177/1479973119869329

**Published:** 2019-08-26

**Authors:** Gill Gilworth, Simon Lewin, Alison J Wright, Stephanie JC Taylor, Rachel Tuffnell, Lauren Hogg, Nicholas S Hopkinson, Sally J Singh, Patrick White

**Affiliations:** 1 Department of Public Health and Primary Care, School of Population Health and Environmental Sciences, King’s College London, London, UK; 2 Norwegian Institute of Public Health, Oslo, Norway; 3 Health Systems Research Unit, South African Medical Research Council, Cape Town, South Africa; 4 Centre for Behaviour Change, Department of Clinical, Educational and Health Psychology, University College London, London, UK; 5 Bart’s and London School of Medicine and Dentistry, Queen Mary University of London, London, UK; 6 The Pulmonary Rehabilitation and Integrated Respiratory Team, King’s College Hospital NHS Foundation Trust, London, UK; 7 Physiotherapy Department, Guy’s and St Thomas’ NHS Foundation Trust, London, UK; 8 National Heart and Lung Institute, Imperial College London, London, UK; 9 Centre for Exercise and Rehabilitation Science, University Hospitals of Leicester NHS Trust, Leicester, UK

**Keywords:** Lay health workers, pulmonary rehabilitation, COPD, patient navigators

## Abstract

Lay health workers (LHWs) can improve access to services and adherence to treatment, as well as promoting self-care and prevention. Their effect in promoting uptake and adherence in pulmonary rehabilitation (PR) for chronic obstructive pulmonary disease (COPD) has not been tested. PR is the most effective treatment for the symptoms and disability of COPD, but this effectiveness is undermined by poor rates of completion. Trained LHWs with COPD, who also have first-hand experience of PR, are well placed to help overcome the documented barriers to its completion. The relationship between LHWs and patients may be one of the keys to their effectiveness but it has been little explored. Semi-structured qualitative interviews were used with the aim of examining the LHW-patient partnership in a feasibility study of trained PR-experienced LHWs used to support COPD patients referred to PR. Twelve volunteers with COPD who completed LHW training supported 66 patients referred for PR. All 12 of these LHWs gave end-of-study interviews, 21 COPD patients supported by LHWs were also interviewed. Patients reported that the LHWs were keen to share their experiences of PR, and that this had a positive impact. The enthusiasm of the LHWs for PR was striking. The common bond between LHWs and patients of having COPD together with the LHWs positive, first-hand experience of PR were dominant and recurring themes in their relationship.

## Introduction

Lay health workers (LHWs), known commonly in the United States as patient navigators or community health workers, can improve access to health services, adherence to treatment and promote self-care and prevention.^1–3^ LHWs are described as usually receiving ‘job-related training but having no formal professional or paraprofessional tertiary education, and can be involved in either paid or voluntary care’.^2^ The use of LHWs in treatment and appointment adherence was first described in the 2000s.^1^ In low-income countries, LHWs or community health workers are frequently used to provide healthcare as substitutes for formally trained staff.^2,4^ In high-income countries, LHWs are used to augment and extend health services as ambassadors or champions of particular health goals including health promotion and prevention.

The effectiveness of LHWs in promoting uptake and adherence in pulmonary rehabilitation (PR) for chronic obstructive pulmonary disease (COPD) has not been tested. PR is the most effective treatment for the symptoms and disability of COPD, the commonest lung disease caused by smoking.^5^ However, the effectiveness of PR in COPD is undermined by poor rates of uptake and completion.^6^ About 40,000 patients with COPD are referred to PR each year in England, less than 3% of those symptomatic with the disease.^7^ Typically, only 40% of these will complete the treatment.^6,8^ Trained LHWs with COPD, who also have first-hand experience of PR, are well placed to help overcome the barriers to its completion.^9^ These barriers include disruption to valued routines, referrers’ uncertainty of the effectiveness of PR, inconvenient timing, travel issues, lack of perceived benefit, being a current smoker and co-morbidities, particularly depression.^8,10^ A recent systematic review found insufficient evidence of interventions to improve the uptake and completion of PR provided.^11^ However, in a qualitative study of people with COPD who had recently completed PR, participants would have welcomed the help of other patients experienced in PR.^12^

Our understanding of the barriers to completion of COPD suggested that the use of trained LHWs with COPD to support COPD patients may offer a key opportunity to improve the uptake and completion of PR. By sharing their own positive experience of PR, for example, they could help counteract referrers’ uncertainty of the effectiveness of PR. They could help overcome attendance barriers through support with journey planning or accompanying individuals to the first assessment and the first PR class. In a feasibility study of using PR-experienced COPD patients, trained as LHWs, to enable other COPD patients to benefit from PR we found that COPD patient volunteers can be recruited, trained, retained and can deliver the intervention with fidelity.^9^ The relationship between LHWs and patients may be one of the keys to their effectiveness but has been little explored. Semi-structured qualitative interviews were used with the aim of examining the LHW-patient partnership in depth. The importance of the LHW-patient relationship has not previously been examined in the context of COPD. However, it seems likely to be an important factor in facilitating treatment adherence.

The LHW intervention underlying this investigation is based on the theory of LHW working elaborated by Gale and incorporates theory-based targeting and tailoring of behaviour change techniques.^13–15^ The aim of this qualitative evaluation was to investigate the experiences of COPD patients referred to PR and supported by trained, PR-experienced volunteer LHWs. We also aimed to understand the motivation of those COPD patients who had themselves undergone PR and volunteered to support others with COPD. Finally, we investigated how acceptable the volunteer LHWs and referred COPD patients found the LHW support system.

## Methods

This study used a qualitative approach, nested in a feasibility study of an LHW intervention to improve the uptake and completion of PR.^9^ The PR programmes to which patient-participants were referred met an international standard of 12–14 sessions over 6–7 weeks.^16^ In the feasibility study, LHWs were recruited from COPD patients who had previously completed PR. The LHWs were trained and then supervised throughout the intervention. Patients referred to the PR service were invited to take part in the study. Their participation included agreement to provide an interview at the end of the study to discuss their experience. All LHW-patient interactions were recorded and subsequently analysed to assess intervention fidelity. The LHW training and supervision, and recording of LHW-patient interactions, are described in detail elsewhere.^9^ The LHW role description which includes a description of the intervention is given in Online Supplementary File. The LHW role was set up as a voluntary role, based on the advice of the project’s COPD patient advisory group. Whenever possible, LHWs were introduced to the patient-participants they supported after referral but before assessment for PR. This had the dual aims of providing patient-participants with support as early as possible and improving attendance at the pre-PR assessment. Eight patient-participants made requests for specific gender LHWs and all were allocated to the LHW gender requested.

Qualitative data for this study were collected using semi-structured interviews with LHWs and patient-participants, supported by topic guides for each group (Tables 1 and 2). The term patient-participant is used to distinguish patients referred to PR from the LHWs who were participants also. The topic guides for the interviews were developed with the support of the project’s COPD patient advisory group. Interviews were facilitated by one of the investigators (GG) who has a background in physiotherapy and is an experienced qualitative researcher. Preliminary analysis of the interview transcripts was undertaken as the interviews progressed.

**Table 1.** LHW interview topic guide.

1. What attracted you about volunteering as a LHW?	
	What were your expectations?
	Probes-things that you thought might be good about getting involved? Any worries?
2. How did you find the process of volunteering – information provided, interview?	
3. How did you find the training as preparation for the LHW role?	
	Probes – what did you find helpful, any gaps?
	Did you feel different by the end compared to at the start of the training? – in what way?
4. How did you find the arrangements for the introduction of the patients you supported?	
5. How did you feel the patient support went?	
	Anything that you can give as examples of things you felt went particularly well, or things that you felt could have gone better?
	How was the first call? How were you feeling?
	How did you find the recording of calls and conversations?
	Did you have to adjust your approach for different patients you supported?
6. Once you were supporting patients what do you think were the key issues that influenced patients’ participation in PR?	
7. How did you find the monthly mentoring meetings?	
8. What do you think about the reimbursement of expenses and the payment for research participation?	
9. If we were going to do this project again what advice would you give us?	

LHW: lay health worker; PR: pulmonary rehabilitation.

**Table 2.** Topic guide for patient-participant interviews.

1. Own recent experience of PR	
	Was this your first-time doing PR? How did you find it?
	What were your feelings about PR when it was offered to you and before starting the classes? How did that change over time?
2. Getting involved in the LHW study	
	Why did you sign up to the LHW project? Was there anything in particular that appealed to you about it?
	What were your expectations? Did you have any concerns about getting LHW support?
3. The LHW support experience	
	Tell me about your LHW and the contact you had with them
	What went well? Was there anything that went less well?
	Did you meet up at all or only speak on the phone? How was the frequency of the contact you had from your point of view?
	Tell me about your feelings about your LHW, for example, what was it like talking to them? How did you feel once they had been in touch with you?
4. Attending PR	
	Was there anything in particular that influenced you going to the PR classes?
	Did your LHW give you any help/support/advice on overcoming any difficulties related to attending PR?
5. Overall what do you think about the idea of LHWs’ involvement in the support of people who have been offered pulmonary rehabilitation?	
	Any suggestions for further research in this area?
6. If you didn’t finish PR, do you have any ideas of other approaches that might have helped you decide to attend PR or to keep going to the classes?	

PR: pulmonary rehabilitation; LHW: lay health worker.

LHWs who had supported patient-participants were invited, at the end of the intervention, to participate in an interview about their experience of the intervention. Patient-participants who were supported by LHWs and who attended the final PR session were given by hand an invitation to a qualitative interview about their experience of the intervention. Invitations were also sent to those patient-participants who did not attend the final session of PR to ensure all of the 66 patient-participants who received LHW support had the opportunity to take part in a qualitative interview. Written informed consent was obtained from all participants.

Face-to-face interviews were conducted with participants in their choice of venue, at home or in another place convenient to them such as a cafe or university meeting room. Interviews were digitally recorded and transcribed verbatim. Follow-up questions and prompts were tailored to the individual participant’s responses with the purpose of clarifying and expanding on areas of importance to the participant or of relevance to the research objectives. Interviews lasted for between 28 and 60 minutes. Ethical approval was provided by the National Research Ethics Service (NRES) Committee, London – Westminster. Research Ethics Committee (REC) reference 14/LO/2313.

## Data collection and analysis

The LHW interview transcripts and the transcripts from the patients they supported (referred to as patient-participants) were analysed as separate data sets. Thematic analysis was undertaken.^17^ Coding of relevant sections, and an iterative process of refining the thematic structure, was completed through reading and rereading the data. GG coded all of the transcripts and then led the coding and analysis. PW coded a sample of 10 transcripts (5 from each data set) for inter-coder verification. The agreed themes formed a coding index used as a means of coding each subsequent transcript. Analysis of patient-participant interviews was conducted alongside data collection to inform subsequent interviews and so that data saturation could be identified. The coding index was constantly refined throughout the process of data analysis as new themes emerged or were merged. The semi-structured style of data collection allowed for a fusion of deductive analysis (with themes derived primarily from topics in the interview guide) while also allowing for themes to emerge directly from the data (inductive analysis).^18^ Data organization was assisted by the use of NVivo software (QSR International, PTY, Victoria, Australia).

## Results

The characteristics of 20 LHWs who commenced training, the 12 who went on to support patients and of the 21 patient-participants who expressed interest and took part in end-of-study interviews are given in Table 3. Fifteen LHWs completed training. Three dropped out after training, one due to illness, one due to family bereavement and one was unable to master use of the smartphone. Twelve LHWs supported patient-participants during the feasibility study, all of whom gave end-of-study interviews. The twelve LHWs supported 66 referred patients with COPD over a period of 10 months. The interviews were guided by a list of topics (Table 2) but participants were encouraged to speak freely about their experiences and to introduce topics for discussion that were important to them.

**Table 3.** Characteristics of volunteer LHWs and of patient-participants.

Characteristics	Volunteers accepted for training (*n* = 20)	LHWs who supported patient-participants (*n* = 12)	Patient-participants (*n* = 21)
Mean age (range)	67.5 (55–79) years	66.2 (55–79) years	69 (43–89) years
Male	11	6	12
Mean time since completing PR (range)	5.5 months (range 0–14)	4.7 months (range 0–12)	n/a
Current smoker	8	5	n/a
Completed PR^a^ Did not complete Did not start	20	12	14 6 1

LHW: lay health worker; PR: pulmonary rehabilitation; n/a: not available.

^a^ From point of referral.

The 21 patient-participants who were interviewed had been supported by 11 different LHWs. None of the patient-participants that were supported by one of the LHWs agreed to an interview. One patient-participant undertook an interview by phone while all other interviews were conducted face-to-face. Data collection was stopped after 21 interviews as no new themes had emerged from the last three transcripts.

### LHW experiences of the patient support intervention

#### Volunteering

The main factors in LHWs’ decisions to volunteer were a desire to help others (*n* = 5), a desire to give something back to the NHS (*n* = 4), experience of helping others during their own PR (*n* = 3) and having spare time (*n* = 3). Five had previous experience of volunteering.

LHWs enthusiasm for PR was striking:

I’ve been so happy to do this, it’s made my life a little bit more enriched and it has fulfilled something in me. (LHW 13)

it helped me, the exercise [PR] helped me…and I wanted to I suppose pass that onto someone else. (LHW 07)

Volunteering and selection for the LHW training included a face-to-face interview with members of the research team (PW and GG). Some of the volunteers found this daunting:

It was a bit nerve-wracking at first…. I did feel like I was back at school…. (LHW 14)

all…, had to be done because we’re working with the National Health Service and we’re going to talk to patients and help patients,…So on, and unless you’re 100% sure how the people are, you couldn’t really send us out to them. (LHW 16)

#### Training

The growth in confidence that the volunteers felt as a result of the training process was an important sub-theme. There was a clear progression from a feeling of trepidation turning into confidence.

…when I first went in there it’s quite daunting with all the people there…at the end of it, yes, I had so much more confidence in meself and I had more feelings to help people and I thought yeah, I can do this. (LHW 16)

I don’t think I’d have been as capable without the training. (LHW 05)

The pace of learning differed among LHWs highlighting the need for additional training for some volunteers. In relation to role play:

It would be good to have a little bit more practice, yeah, especially for someone like me who’s a little bit apprehensive…. (LHW 14)

#### LHWs’ experiences of supporting patient-participants

Some of the LHWs were apprehensive before making the first phone calls to people but reported that the introductions went well:

It’s like when you get in a car to drive it for the first time, you’re nervous……and it’s the same thing, when you phone them, you sort of say, ‘Now I must remember to introduce myself properly. (LHW 09)

There was a range of views on the advantages and disadvantages of phone support compared to meeting up with patient-participants.

I just like to watch their reactions, their body language and that…and know that I’m gonna get on with them, you know, that’s what I like about a face-to-face. (LHW 15)

I didn’t want to be just somebody on the end of a phone saying ‘oh no, it will be much better when you’ve done your exercise’,…I mean it suits some people, it doesn’t suit other people. (LHW 20)

The common ‘*bond*’ of having COPD, and the LHWs’ positive, first-hand experience of PR were seen as important in building relationships with patient-participants:

we’ve got that bond straight away. I can relate to their breathing issues, you know. (LHW 04)

she was very grateful that she’s actually met someone with the same condition that she’s got,…I don’t know, we’ve sort of formed a bond, you know…this has brought us together. (LHW 15)

#### The role of mentoring meetings

Eight mentoring meetings were held during the period in which LHWs supported patient-participants. The meetings were facilitated by an independent mentor. LHWs suggested that they would have felt isolated without these meetings:

it’s quite good having the meetings…because otherwise you’d be very much on your own just making the calls. You wouldn’t know what’s going on,…I think you want to meet the other people that are doing it. (LHW 13)

At the mentoring meetings, LHWs shared their experiences of supporting patient-participants. These meetings had an important social function; however, there was a desire for the meetings to be focused more on the LHW role and problem-solving:

I found a lot, a lot of talk that was going on at the meetings wasn’t actually relevant…it just needs a little bit more direction…if you’ve got any problems I’d actually be better off talking to another lay health worker because they’ve actually done it and ask a few lay health workers what they think about a particular problem…so yeah, I think it’s a good idea. (LHW 6)

### Patient-participant experiences of the intervention

#### Contact frequency

The LHWs engaged to different degrees with patients: some patients had very little contact with their LHW while others had up to 20 contacts by phone and face-to-face over a 2–3-month period:

[I] had an initial phone call but no further contact with the lay health worker…[I] like the idea but lay health workers need to be monitored to make sure they’re ringing patients when they say they are. (Pt 74)

Two themes emerged relating to the LHW-patient relationship; firstly, observations on the LHW support and secondly the LHW as a person. Each of these broad themes had a number of sub-themes shown in Table 4.

**Table 4.** Patient-participant sub-themes: ‘The LHW support system’ and ‘LHW as a person’.

The LHW support system	The LHW as a person
Sub-themes: Supportive ‘perks you up’ LHW sharing own experience of PR Made the difference to me going Timing, type and frequency of contacts A common bond (they have COPD too) Lack of contact ‘I could have done with more support’	Sub-themes: Easy to talk to A good listener ‘Knew what they were talking about’ (so I could trust them) ‘I thought they would be more chatty’

LHW: lay health worker; PR: pulmonary rehabilitation; COPD: chronic obstructive pulmonary disease.

#### The LHW support system

Most participants talked positively about the support from their LHW:

it made me want to go more and if I was feeling a bit depressed or don’t want to go,…it just needs a phone call to build somebody’s spirit back up. (Pt 090)

Some talked about the benefits of being able to meet with their LHW at the PR appointments:

it was nice to have her support because she came the first time and she came the second time. (Pt 032)

Patients noted that the LHWs were keen to share their experiences of PR, and that this had a positive impact:

Interviewer: ‘…. what you were feeling at that time about the PR?’

Patient-participant: ‘Stressed…yeah, but I must have had 3 or 4 phone calls, she (LHW) convinced me otherwise’. (P002)

For some patient-participants LHW support was crucial to their attendance:

…I did say to J (name of LHW) I mean if you didn’t come with me today I would never come I’m not going to lie you know and she said ‘oh I gathered'

Whenever possible, patients were put in touch with their LHW before their first assessment. Support on the day of the first assessment was received positively. It became apparent that the frequency of contacts was variable and, in some cases, patients would have appreciated more LHW input:

it would have been good to have a bit more support, especially at the beginning because I found it hard…. I would have been happy to meet with him more than once, or to talk to him on the phone a bit more but he hardly ever rang me. (Pt 061)

Some patients did like to meet with their LHW in order to ‘put a face to a name’, but others were happy for all interactions to be by phone:

Yes, yeah I did choose that way because she said ‘you know we could meet if you want, you don’t have to’…‘no, that would be nice’ because it’s nice to put a face to a voice, you can do more face to face that’s not so very good with the phone. (Pt 072)

The benefits of the common bond of a shared disease, and LHWs’ experience of the treatment that they were promoting, were viewed by both patient-participants and LHWs as important elements of the patient-LHW relationship:

So it’s good to have somebody supporting you that has got the same kind of condition as you have because then you can relate to it and they can feel how you feel. (Pt 005)

I think it’s a very good knowing you’ve got someone who understands how you, what you’re going through…I feel you’re free to say what you want. (Pt 19)

#### The LHW as a person

Patients described their LHWs in largely complimentary terms including kind, caring, laid back, genuine, polite, down to earth, jolly and helpful. The experience and knowledge of the LHWs was valued and this helped to foster trust between the patient and LHW:

She was genuine and…she knew what she was talking about and she knew what she could do and she did it with sincerity…I give her 101 out of 100. (Pt 123)

She knew what she was talking about actually. (Pt 002)

I don’t really remember asking her anything that she didn’t know about. (Pt 038)

There were also patient-participants who commented that they would have liked to speak to their LHW for longer:

I thought I might get someone a bit more chatty but when he rung me he just said when are you starting? He didn’t really say much else, he didn’t chat for very long. (Pt 061)

## Discussion

This study illustrates the experiences of LHWs and of the patient-participants they supported in the LHW intervention to improve the uptake and completion of PR for COPD. The findings suggest that the support provided by LHWs impacted positively on patient-participants who appreciated the buddying and reassurance provided by LHWs. In particular, they valued early contact with LHWs and being accompanied to the first assessment and the first PR class, when appropriate. Consequently, the optimum time point for LHWs to be deployed appears to be prior to the initial assessment for PR. The shared experience that LHWs and patient-participants had of COPD and of PR was a key part of this supportive relationship. Patient-participants felt that the LHWs understood what they were going through. Some patient-participants were disappointed with the limited contact they had at a time when they would have wanted to have more.

Overall, both LHWs and patient-participants seemed to find the intervention acceptable. These findings are congruent with those from other parts of the project which showed the feasibility of the intervention.^9^ They offer some explanatory potential for the underlying theory and mechanism of the LHW intervention in promoting PR.^13^ Based on advice from the project’s COPD patient advisory group, the LHWs in this study worked as volunteers. This puts the intervention into an LHW category marked by altruism.^19^

Among the lessons learnt with respect to the LHW intervention was the need for regular monitoring of the LHWs, as they supported patient-participants, to optimize availability and contact and to promote the combination of telephone and face-to-face contact. Some LHWs may have needed more time in training, with greater attention to repetition of role play exercises.

This study is unusual in having conducted a qualitative examination of the experience of both LHWs and patient-participants and of the relationships between them from the perspective of both groups over the same period of an intervention. In addition, the LHWs and patients shared the same progressive and limiting disease, the same specific treatment method and the same locality. The concept of the LHW as a substitute for professional healthcare workers or as ambassadors or champions of particular health goals places emphasis on the specialist knowledge or skill base of the LHW, for example, ability to take and interpret blood pressure or ability to give health promotional or preventative advice.^1,2,4^ The model we have developed places more emphasis on communication skills, the identification of barriers to treatment, the use of behaviour change techniques to address those barriers and the conduct of a one-to-one relationship through which trust is built.^9^ It is this latter element which was the chief focus of this qualitative analysis. The potential advantage of shared concerns between LHWs and patients and the personal characteristics of trustworthiness, respect, kindness and empathy have been noted in many LHW studies.^20^ They have been considered instrumental in improving the uptake of services and enhancing health outcomes.

Active support by health services of the LHW programme, as in the PR service in this research, can lead recipients to view the LHWs as legitimate and credible and to view their services as relevant and valuable.^20^ This also provides LHWs with social recognition and empowerment. These factors can, in turn, lead to good relationships between LHWs and recipients and can also increase the willingness and ability of LHWs to deliver services.

The strengths of this study are to do with its novelty, its contribution to the growing field of LHWs and patient navigators and the link between LHWs and patients through their shared illness, the treatment and their common location. The common ground between LHW and patient is likely to be a good basis for exploring obstacles to attending PR and for seeking solutions to those obstacles. Patient-participants confirmed this element. All of the LHWs who were invited took part in the interviews. This may be partly due to the contract between them and the research team, but their enthusiasm and commitment was also a factor.

The limitations of this research include those found in any qualitative study in that we can’t be sure that the patient-participants’ views we obtained represented the views and perspectives of all patients referred to PR. We were reassured that we obtained no new data themes after the 18th person interviewed. Patient-participants who consented to participate in the study but did not attend PR, or withdrew early from the treatment, may have been less likely to take up the invitation to be interviewed. Patients with more severe disease may not have volunteered for interview and some patients may not have felt able to criticize the LHW with whom they had worked, given that they were volunteers who were ‘doing their best’.

## Conclusion

This research was conducted as part of a successful feasibility study for a clinical trial of LHWs to improve uptake and completion of PR. The feasibility of recruiting, training and retaining PR-experienced LHW volunteers has been reported.^9^ In addition, this qualitative study has highlighted the value placed by both LHWs and patient-participants on the relationship between them. In describing the common bond that arose from their shared disease and treatment, the LHWs and patient-participants have emphasized the importance of that element of the intervention to its success.

## Supplemental material

Supplementary_file_-_Lay_health_worker_role_description - The lay health worker–patient relationship in promoting pulmonary rehabilitation (PR) in COPD: What makes it work? Click here for additional data file. Supplementary_file_-_Lay_health_worker_role_description for The lay health worker–patient relationship in promoting pulmonary rehabilitation (PR) in COPD: What makes it work? by Gill Gilworth, Simon Lewin, Alison J Wright, Stephanie JC Taylor, Rachel Tuffnell, Lauren Hogg, Nicholas S Hopkinson, Sally J Singh and Patrick White in Chronic Respiratory Disease
