# Supplementary material for: The lay health worker–patient relationship in promoting pulmonary rehabilitation (PR) in COPD: What makes it work?
Source: Chron Respir Dis. 2019 Aug 26;16:1479973119869329. doi: 10.1177/1479973119869329 (PMC6710699; doi:10.1177/1479973119869329)
Supplement: Supplementary_file_-_Lay_health_worker_role_description - The lay health worker–patient relationship in promoting pulmonary rehabilitation (PR) in COPD: What makes it work? [file Supplementary_file_-_Lay_health_worker_role_description.pdf]

a) Role Description for Lay Health Worker

Responsible Organisation: King's College London, Division of Health and Social Care Research.

Role Title: Lay health worker in pulmonary rehabilitation.

Responsible to: Dr Patrick White, Reader in Primary Care Respiratory Medicine.

Purpose / Summary of the role

To assist COPD patients newly referred to Pulmonary Rehabilitation in attending the course.

Description of tasks

1. To agree to support up to eight patients referred to pulmonary rehabilitation over a period of up to twelve months.
2. To attend three training sessions in the role of lay health worker.
3. To support the patient using the understanding and skills acquired at the training sessions.
4. To speak with, meet and, if desired, accompany the patient to pulmonary rehabilitation.
5. To make a digital recording, using equipment provided by the research team, of conversations with patients to help with evaluation of this project.
6. To attend meetings with other lay health workers arranged by a professional mentor. The purpose of the mentor meetings is to review the role of lay health worker. Lay health workers will be able to share experiences, to learn from each other, and to solve problems that arise.
7. To treat all personal information given by patients in complete confidence.
8. To inform the research team of any concern or worry about the lay health worker's own welfare that arises in the course of the volunteering role.
9. To inform the research team of any concern or worry about the welfare of any patients that arises in the course of the volunteering role.
10. To provide an interview to the research team to evaluate the role of being a lay health worker.

Time commitment:

To agree to act as a volunteer for up to one year.

To attend three training sessions in East Dulwich.

To provide support for up to 8 patients, 2 months at a time (with some overlap) over a year.

To speak on the telephone with each patient no more than eight times. Each telephone conversation to take no more than thirty minutes.

To meet with each patient at least once and no more than four times. Each meeting to last no more than three hours.

To attend at least four mentoring meetings (1½ hours max each) in the course of the year.

Skills and Qualifications:
Previous experience of at least one complete course of Pulmonary Rehabilitation for COPD.
Ability to speak, read and write in English.
Ability to use a telephone.
Ability to travel independently in south London.

IMPORTANT NOTE: All volunteer lay health workers must undergo a Disclosure and Barring Service (DBS) check, previously known as a Criminal Record Bureau check. We will provide more information on this at interview.

Training and support:

Training will be provided by the Royal Society of Public Health at Dulwich Community Hospital on three days (1 day a week for 3 weeks). Each session will be from 10.30 am – 3.30pm. We will provide a mentor for lay health workers who will meet with them in a group and support them in their work with patients.

Reimbursement of expenses:
Travel and subsistence will be reimbursed, each at a set rate to be agreed.

Payment for research work:
The volunteer will be paid for activities related to the research evaluation. These will include a payment of up to £60 per patient supported for the recording of conversations with patients and a one-off payment of £50 for providing a research interview. Lay health workers who support 8 patients will be paid £480 for their research contribution in recording interviews.

Benefits to volunteer:
Volunteering as a Lay Health Workers is an opportunity to support other COPD patients to benefit from the PR service. We hope the volunteer will find this role enjoyable and fulfilling. We think it will prove extremely valuable to patients. This is the first time support through lay health workers has been attempted in this setting.

o0o
